# Compassion Satisfaction, Burnout, and Compassion Fatigue in Cardiac Physicians Working in Tertiary Care Cardiac Hospitals in Pakistan

**DOI:** 10.7759/cureus.3416

**Published:** 2018-10-05

**Authors:** Haider Ghazanfar, Muhammad Tariq Chaudhry, Zameer Ul Asar, Usama Zahid

**Affiliations:** 1 Internal Medicine, Shifa College of Medicine, Islamabad, PAK; 2 Epidemiology and Public Health, Combined Military Hospital, Peshawar, PAK; 3 Cardiology, Punjab Institute of Cardiology, Lahore, PAK; 4 Cardiac/thoracic/vascular Surgery, Shaikh Zayed Hospital, Lahore, PAK

**Keywords:** compassion fatigue, secondary traumatic stress, burnout, compassion satisfaction, cardiac caregiver

## Abstract

Introduction

The purpose of this study is to determine the prevalence and risk factors of compassion satisfaction, compassion fatigue, and burnout among cardiac physicians working in tertiary care cardiac hospitals in Pakistan.

Materials and methods

We performed a cross-sectional study in four tertiary care cardiac hospitals located in Rawalpindi and Lahore, Pakistan from June 2017 to January 2018. The study comprised of three stages. The first stage involved administration of the Professional Quality of Life Scale (ProQOL-5) questionnaire in order to assess the prevalence of compassion satisfaction, compassion fatigue, and burnout in cardiac physicians. In the second stage, cardiac physicians were divided into two groups according to their compassion fatigue level. In the third stage, 50 participants were selected via convenience sampling to participate in a 15-minute interview regarding compassion fatigue and risk factors. The data obtained was analyzed using the Statistical Package for the Social Sciences (SPSS), version 21.0 (IBM, Armonk, NY).

Results

The mean age of the participants was found to be 39.2 ± 6.3 years. Out of the 200 participants, 110 (55.0%) were males while 90 (45.0%) were females. The mean score in the compassion satisfaction category was 39.13 ± 5.54 while the mean score of burnout category was 24.7 ± 4.28 and that of secondary traumatic stress (compassion fatigue) was 25.97 ± 6.39. Participants whose age was less than 40 years had a higher score in Burnout (p < 0.001) and secondary traumatic stress category (p < 0.05).

Conclusions

In Pakistan compassion fatigue, despite being reported as a negative phenomenon, has received little or no attention. There is a dire need to increase awareness about compassion fatigue and burnout among cardiac care physicians in Pakistan.

## Introduction

Working with patients suffering from cardiac diseases exposes cardiac physicians to their patient's consistent pain and suffering which can be a potential source of his emotional and psychological stress. Cardiac physicians spend a lot of time caring for their patients in cardiac health care settings. They provide health care services to cardiac patients by addressing a variety of acute and chronic health care conditions. This all can have devastating impacts on cardiac physicians’ quality of life by exposing them to the extreme pain and suffering of patients and their families.

Compassion fatigue is an adverse outcome that physicians may experience as a result of their work with ill people. Compassion fatigue is the state of emotional, biological and physiology exhaustion due to constant exposure to the suffering of others over a prolonged duration of time and is characterized by a gradual lessening of compassion over a passage of time [1]. Health care professionals are at a higher risk of suffering from compassion fatigue [2]. According to a study performed by emergency department nurses, 86% of the participants had a moderate to high level of compassion fatigue [3]. If remain unchecked, compassion fatigue can lead to disastrous consequences [4].

Compassion fatigue is associated with a low level of compassion satisfaction and a high level of secondary traumatic stress (STS). Compassion fatigue is different from burnout as burnout is related to chronic overwork without any extreme or traumatic stressful events while compassion fatigue is directly linked to exposure of extremely stressful situations over a passage of time [5-6].

Various studies have concluded that clinicians who care for seriously ill patients are at higher risk of developing compassion fatigue as compared to clinicians who deal with patients who are not in critical condition [7]. Cardiac physicians are continuously exposed to serious or critically ill patients throughout their careers and are thus at a higher risk of developing compassion fatigue. Despite the importance of this issue very limited work has been done on the prevalence and associated risk factor of compassion fatigue in cardiac physicians, especially in Pakistan. The purpose of this study is to determine the prevalence of compassion satisfaction, compassion fatigue, and burnout among cardiac physicians working in tertiary cardiac hospitals in Pakistan.

## Materials and methods

We performed a cross-sectional study in four tertiary care cardiac hospitals located in Rawalpindi and Lahore, Pakistan from June 2017 to January 2018. The hospitals were selected in three stages. The first stage involved the creation of a sampling frame which comprised of all the major tertiary cardiac hospitals in Rawalpindi and Lahore. A number was assigned to all the hospitals in the sampling frame. In the second stage, two hospitals were selected randomly via the help of computer software in order to ensure that each hospital had an equal chance of selection. The participants were selected through convenience sampling. All cardiac physicians and surgeons working in the hospital and who did not meet the exclusion criteria were eligible for the study. The data was not obtained from cardiac physicians and surgeons who were working in the hospital for less than three months or had associated psychiatric illness or had a recent history of emotional stress like a family breakup, having a special child or exposure to severe trauma. Informed consent was taken from all the participants. Permission was also taken from the institutional ethics committees of the participating hospital.

The study comprised of three stages. The first stage involved administration of the Professional Quality of Life Scale (ProQOL-5) questionnaire in order to assess the prevalence of compassion satisfaction, compassion fatigue, and burnout in cardiac physicians. ProQOL-5 comprised of 30 questions [8]. The ProQOL-5 responses are divided into three separate 10-item subscales which are compassion satisfaction, burnout and secondary traumatic stress. Each subscale is scored separately. For each subscale, the total score is reported as low, average or high. A higher score in compassion satisfaction component represents a great ability of the person to be an effective care provider while a higher score in burnout component and secondary traumatic stress is associated with a higher risk of developing burnout and compassion fatigue, respectively.

Each participant was given sufficient time to complete the questionnaire. In the second stage, cardiac physicians were divided into two groups based on their compassion fatigue level. Cardiac physicians with the high and average level of compassion fatigue were labeled as participants with high compassion fatigue level while cardiac physicians with low level of compassion fatigue were labeled as participants with low compassion fatigue level. The secondary traumatic stress subscale is a good indicator of compassion fatigue and has been used to assess compassion fatigue in various studies [9]. The ProQOL-5 has been extensively tested and has been found to be reliable and valid [3]. In the third stage, 50 participants were selected via convenience sampling to participate in a 15-minute interview regarding compassion fatigue and risk factors. The data obtained was analyzed using the Statistical Package for the Social Sciences (SPSS), version 21.0 (IBM, Armonk, NY). Independent-samples t-test was used to compare the various groups with subscale category. P-value < 0.05 was considered as statistical significant.

## Results

The mean age of the participants was found to be 39.2 ± 6.3 years. Out of the 200 participants, 110 (55.0%) were males. The majority (82.5%) of the participants were currently married. About 112 (56.0%) of the participants were working as cardiac physicians for as less as five years. A majority (55.5%) of the participants worked for less than eight hours a day. About 45.5% of the participants saw less than 30 patients every day. Only 14 (7%) of the participants were smokers. About 63.5% of the participants slept for less than six hours.

The level of compassion satisfaction, burnout and secondary traumatic stress (compassion fatigue) among the participant have been mentioned in Table 1.

**Table 1:** The Level of Compassion Satisfaction, Burnout and Secondary Traumatic Stress.

	Level of each subscale category		
Subscale category	High	Average	Low
Compassion Satisfaction	32.0%	68.0%	0 %
Burnout	0%	64.5%	35.5%
Secondary Traumatic Stress (Compassion Fatigue)	0%	58.0%	42.0%

None of the participants had a high level of burnout and secondary traumatic stress (compassion fatigue). The mean score in the compassion satisfaction category was 39.13 ± 5.54 while the mean score of burnout category was 24.7 ± 4.28 and that of secondary traumatic stress was 25.97 ± 6.39. This has been presented in Table 2.

**Table 2:** Mean Score of Sub-scale Categories.

Subscale category	Mean ± SD
Compassion Satisfaction	39.13 ± 5.54
Burnout	24.7 ± 4.28
Secondary Traumatic Stress (Compassion Fatigue)	25.97 ± 6.39

Cardiac physicians with a high and average level of compassion fatigue were labeled as participants with high compassion fatigue level while cardiac physicians with a low level of compassion fatigue were labeled as participants with low compassion fatigue level. Independent-samples t-test was used to compare the groups with various demographic variables. Male participants had a significantly higher score in the Compassion Satisfaction Category (p < 0.05). There was no significant difference in the burnout category and secondary traumatic stress category between male and female participants (p > 0.05). Participants who worked more than eight hours had a significantly higher score in the Compassion Satisfaction Category (p < 0.001). None of the subscale categories was significantly associated with the amount of patient seen by the participant (p > 0.05). Participants with age greater than 40 years had a significantly higher score in the Compassion Satisfaction Category (p < 0.001) as compared to participants whose age was less than 40 years. Participants whose age was less than 40 years had a higher score in burnout (p < 0.001) and secondary traumatic stress category (p < 0.05).

Participants who had slept for less than six hours had a significantly higher score in burnout component and secondary traumatic stress as compared to participants who had slept for more than six hours (p < 0.05). Participants who were married had a higher score in burnout component and secondary traumatic stress component (p < 0.001).

Analysis of the response given by the participant in the 15 minutes interview revealed:

1)     Institutional processes like performance-oriented appraisals based on workload management and patients’ feedback are the strongest forecasters of emotional exhaustion among cardiac caregivers.

2)     Cardiac caregivers are unable to meet their professional and personal obligations toward the organization so work avoidance and attrition are likely outcomes.

3)     Poor working conditions significantly contribute towards compassion fatigue which includes the quality of helping staff, hospital ambiance and the equipment available. Financial dissatisfaction is quite significantly associated with compassion fatigue.

4)     Affected caregivers have a feeling of incompetency thus reduced performance and relatively impaired ability to make treatment decisions for patients.

5)     Victims of compassion fatigue have problems in personal relationships, therefore, they have poor relations with their colleagues and are cynical towards their clients.

6)     More experienced cardiac caregivers feel protected and were at a lesser risk of developing compassion fatigue. Similarly, perceived support of administration seems to be a protective factor. It looks worthwhile for the administration to be perceived as supportive in addition to being supportive as such.

## Discussion

**Figure 1:**
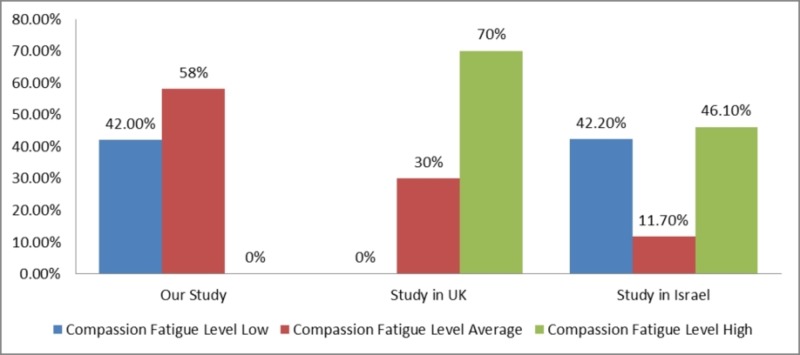
Comparison of Compassion Fatigue Scores.

In our study, 116 (58.0%) of the participants had an average level of compassion fatigue while 84 (42.0%) of the participants had a low level of compassion fatigue. Compassion fatigue in our study was found to be much lower than compassion fatigue found among other studies [10-11]. The high-level category is non-existent in our study as compared to the study done in the UK [10] and Israel [11]. This has been presented in Figure 1.

According to a study done among physician in Israel concluded that female physicians were more prone to develop burnout [10]. Another study done on nurses had a similar conclusion (p = 0.001) [12]. In our study, there was no significant association of gender with any component.

Younger physicians were more likely to develop burnout and compassion fatigue. A possible reason for this finding could be lack of experience in coping with various kinds of work-related stressors. According to a study, newer nurses were more likely to develop compassion fatigue (p < 0.01) and burnout (p = 0.02) as compared to experienced nurses because of lack of prior exposure to the stressful situation [13]. Another reason for this difference could be the extensive clinical experience the older physicians have to attain which endorses a higher level of compassion satisfaction and thus decrease compassion fatigue [14].

A study done on US healthcare workers concluded that healthcare workers who slept for a longer time were less likely to have burnout as compared to healthcare workers who slept for a shorter duration of time [15]. This could be because of the directly proportional association of lack of sleep with burnout.

The single most important contribution of this study is to boost awareness about compassion fatigue and its injurious effects on physicians' health. Continuing medical education [16], ongoing wellness programs [17], regular professional supervision, awareness campaign, regular recreational activities, initiation of compassion fatigue intervention program, development compassion fatigue cell and institutional reforms can have a significant impact on decreasing the prevalence of compassion fatigue. Physician wellness programs have been implanted in various countries to decrease burnout and compassion fatigue among physicians [18].

The small sample size and limited diversity of the sample population was a limitation to the current study. Surveying a larger number of cardiac physicians from healthcare institutions could provide more generalizable information about the prevalence of compassion fatigue and burnout among cardiac physicians. Since the ProQOL-5 survey tool is designed to know the level of compassion fatigue at one point in time, a qualitative approach could be helpful in understanding cardiac physicians' perception of compassion fatigue. A large-scale study of compassion fatigue among cardiac nurses could provide rich and valuable data on compassion fatigue. Also, if a certain population of cardiac physicians consistently score low on compassion fatigue, researchers could investigate what kind of protective measures are being taken by the hospitals to promote cardiac physician health.

## Conclusions

In Pakistan compassion fatigue, despite being reported as a negative phenomenon, has received little or no attention. There is a dire need to increase awareness about compassion fatigue and burnout among cardiac care physicians in Pakistan. This, in turn, will ensure that the patients are provided with the best possible care.

## References

[1] Kase SM, Waldman ED, Weintraub AS (2018). A cross-sectional pilot study of compassion fatigue, burnout, and compassion satisfaction in pediatric palliative care providers in the United States. Palliat Support Care.

[2] van Mol MM, Kompanje EJ, Benoit DD, Bakker J, Nijkamp MD (2015). The prevalence of compassion fatigue and burnout among healthcare professionals in intensive care units: a systematic review. PLoS One.

[3] Hooper C, Craig J, Janvrin DR, Wetsel MA, Reimels E (2010). Compassion satisfaction, burnout, and compassion fatigue among emergency nurses compared with nurses in other selected inpatient specialties. J Emerg Nurs.

[4] Maytum JC, Heiman MB, Garwick AW (2004). Compassion fatigue and burnout in nurses who work with children with chronic conditions and their families. J Pediatr Health Care.

[5] Kleiner S, Wallace JE (2017). Oncologist burnout and compassion fatigue: investigating time pressure at work as a predictor and the mediating role of work-family conflict. BMC Health Serv Res.

[6] Hinderer KA, VonRueden KT, Friedmann E (2014). Burnout, compassion fatigue, compassion satisfaction, and secondary traumatic stress in trauma nurses. J Trauma Nurs.

[7] Lemaire JB, Wallace JE, Dinsmore K, Lewin AM, Ghali WA, Roberts D (2010). Physician nutrition and cognition during work hours: effect of a nutrition based intervention. BMC Health Serv Res.

[8] (2018). The ProQol measure in english and non-english translations. https://proqol.org/ProQol_Test.html.

[9] Lauvrud C, Nonstad K, Palmstierna T (2009). Occurrence of post traumatic stress symptoms and their relationship to professional quality of life (ProQoL) in nursing staff at a forensic psychiatric security unit: a cross-sectional study. Health Qual Life Outcomes.

[10] Sodeke-Gregson EA, Holttum S, Billings J (2013). Compassion satisfaction, burnout, and secondary traumatic stress in UK therapists who work with adult trauma clients. Eur J Psychotraumatol.

[11] El-Bar N, Levy A, Wald HS, Biderman A (2013). Compassion fatigue, burnout and compassion satisfaction among family physicians in the Negev area - a cross-sectional study. Isr J Health Policy Res.

[12] Mooney C, Fetter K, Gross BW, Rinehart C, Lynch C, Rogers FB (2017). A preliminary analysis of compassion satisfaction and compassion fatigue with considerations for nursing unit specialization and demographic factors. J Trauma Nurs.

[13] Kolthoff KL, Hickman SE (2017). Compassion fatigue among nurses working with older adults. Geriatr Nurs.

[14] Craig CD, Sprang G (2010). Compassion satisfaction, compassion fatigue, and burnout in a national sample of trauma treatment therapists. Anxiety Stress Coping.

[15] Smart D, English A, James J, Wilson M, Daratha KB, Childers B, Magera C (2014). Compassion fatigue and satisfaction: a cross-sectional survey among US healthcare workers. Nurs Health Sci.

[16] Krasner MS, Epstein RM, Beckman H, Suchman AL, Chapman B, Mooney CJ, Quill TE (2009). Association of an educational program in mindful communication with burnout, empathy, and attitudes among primary care physicians. JAMA.

[17] Sood A, Sharma V, Schroeder DR, Gorman B (2014). Stress management and resiliency training (SMART) program among department of radiology faculty: a pilot randomized clinical trial. Explore (NY).

[18] Wallace JE, Lemaire JB, Ghali WA (2009). Physician wellness: a missing quality indicator. Lancet.

